# New alternative proposal in physical education: Touchtennis as a racket sport in schools

**DOI:** 10.3389/fspor.2025.1545994

**Published:** 2025-03-03

**Authors:** Paula Teresa Morales-Campo, Salvador Pérez-Muñoz, Sergio López-García, Pelayo Díez-Fernández, Alberto Rodríguez-Cayetano

**Affiliations:** Faculty of Education, Pontifical University of Salamanca, Salamanca, Spain

**Keywords:** racquet sport, didactic intervention, alternative sport, Touchtennis, physical activity

## Abstract

In the last decade, the subject of Physical Education has undergone significant transformations, exploring new teaching alternatives for students to acquire interest and enthusiasm for sport. The main objective of this descriptive study is to bring Touchtennis to schools as an innovative and alternative racket sport, which has attracted interest in the sports field. In terms of methodology, an intervention proposal is presented, designed for the subject of Physical Education, through a methodological sequence, in order to get to know the basic contents of the sport and to improve the physical, psychological, social and emotional components of the student. To date, Touchtennis lacks scientific evidence to support its effects. Consequently, a pedagogical proposal is presented in order to open up a promising field for future research, which could reveal its potential in the educational and sporting sphere. It is therefore essential that, on the basis of this research, empirical tests are carried out to analyse the effect of this sport.

## 1 Introduction

Within the current educational context, the subject of Physical Education must incorporate high quality programmes in order to acquire significant learning and encourage the regular practice of physical activity, emphasising the importance of the health and physical well-being of students (1, 2). In fact, there are many studies that have shown that the practice of exercise or physical activity leads to an improvement in students’ academic performance, which reaffirms that the subject of Physical Education should be a fundamental pillar in people's education (3–6).

From this perspective, it is relevant to highlight the role of teachers in teaching spaces, as they must motivate schoolchildren to practice sport by employing different methodologies and attractive and innovative activities that allow for greater commitment and dedication to practice (7–9). In this line, different educational models oriented towards constructivism have emerged significantly, leaving conventional teaching behind (10). These avant-garde approaches are incorporated into the contemporary educational framework to improve the quality of the teaching process and increase motivation towards the subject, with the teacher being the guide and facilitator of the learning process and the student being the protagonist of his or her own learning (11).

In this sense, the teacher must opt to use more innovative methodologies and practices for teaching content and acquiring competences (12, 13), with the aim of increasing the interest and enthusiasm of the student towards the subject. In relation to new practices, Spanish educational legislation has undergone several adaptations in curricular elements, highlighting the incorporation of alternative sports as content within the subject of Physical Education (14).

While it is true that alternative sports or games are understood as those attractive and novel recreational practices that favour the complete and integral development of students (15, 16) and provide improvements in motor skills (17). It is also stated that they experience numerous advantages, as they increase motivation and enjoyment towards the subject, create bonds of friendship (18), acquire positive values and behaviours (19), develop an inclusive and equal understanding (20), foster cooperation and decision-making, and provide alternative and different practices to the traditional ones (21). In this way, it can be inferred that these types of sports, which are totally recent and innovative, represent a new vision within the Physical Education community, due to their wide variety of characteristics, multiple benefits and the facilities they provide when adapted to different contexts (22).

In this context, racket sports are progressively appearing in teaching plans (23) as an educational potential that not only provides a great motor richness (24), but also improves the perceptive, affective and social components of the schoolchild (25, 26). In fact, Varea (27) stated that, through practice, they improve observation skills, foster understanding and decision-making at a personal and group level, promote education in values and strengthen relationships between peers, creating a positive climate that encourages continuous learning.

Thanks to their great popularity in recent years, they are currently positioned as a relevant and alternative teaching content in education (28). In this regard, Castellar et al. (29), highlighted them as a pedagogical option for their development in the subject of Physical Education. However, the school situation reveals that not all racket sports are implemented by specialist teachers, due to a lack of theoretical/practical knowledge about the sport itself, or the practice of racket sports that are better known or easier to adapt to the teaching environment (30). In this sense, it is recommended that teachers be trained in this sport modality, as its implementation can benefit and improve the quality of teaching (31). Furthermore, Quintas et al. (32) stress that it is necessary for teachers to be trained in new disciplines and emerging trends to enrich teaching programmes and incorporate more varied and up-to-date experiences.

However, in order to teach these sports at school, it is essential to apply the game-based methodology (33). In fact, Wilson (34) has promoted this methodology, given that it provides multiple benefits for the overall learning of schoolchildren. In particular, his philosophy is not based on teaching basic and directed strokes as was the case with traditional teachings (35), but seeks to foster technical and tactical learning through a structured and well-organised gradual progress, in order to perform game-based performances that provide greater efficiency (36). Therefore, the game becomes a valuable tool for the teacher due to its great capacity to promote a more dynamic and motivating environment (37).

In addition to the above, Villamizar (38) indicated that teaching through play is a methodology that is based on the active participation of the student through exploration and discovery, linked to fun and interest in practice. In fact, Carrillo-Ojeda et al. (39) found that learning and play are closely related. Thus, play-based teaching is an essential methodology, as its application demonstrates an improvement in physical, cognitive, social and emotional aspects, facilitating an integral development in the student.

Another pedagogical model used in current teaching is the comprehensive model or Teaching Games For Understanding (TGFU) (40–42). Specifically, this methodology is based on the comprehensive teaching of the game through real situations, where the player learns to make decisions generating more effective results (43). In this way, tactical awareness is a determining factor (44), since knowing only the learning of technique is not enough to be effective in the game (45). Therefore, the TGFU focuses on constructivist learning that favours cognitive aspects through the teaching of game tactics in particular combined (46, 47).

Therefore, the main objective of this research work is to provide the educational community with a pedagogical intervention proposal based on a racket sport, called Touchtennis, in order to inculcate this sport in educational centres in a novel and transformative way, using the game as an educational resource.

## 2 Pedagogical framework

Over time, the educational field has undergone several adaptations in the curricular elements, highlighting the incorporation of alternative or emerging sports as teaching content within the area of Physical Education (14, 18).

From the Spanish legislative point of view, Royal Decree (48), specifically cites softball, pallados, ultimate and pickleball, as sports considered as alternative, as examples of sports that could be implemented in the classroom. Similarly, there are several Autonomous Regional Decrees that set out this need, such as the Decree of the Region of Murcia (49) and the Decree of the Principality of Asturias (50), among others.

In addition to the above, the Order of the Autonomous Community of Aragon (51), refers to the following alternative sports: touchball, indiaka, dodgeball, kin-ball, colphol and korfball. All of them are characterised as alternative or emerging.

In this respect, the Decree of Castilla y León (52), refers to alternative sports or games from the third year of Primary Education onwards. For its teaching, it is necessary to apply pedagogical methodologies adapted to the contents and the particular needs of each group, allowing a competent and quality development during the student's learning process (53). In this sense, León-Díaz et al. (12) and Sierra-Díaz et al. (54) pointed out that the methodological strategies that are implemented must be adapted to the particularities of each environment, that is, they must be adapted to the characteristics of the student, the materials and facilities available and especially to the curricular elements of the educational law. In addition, they stated that these methodologies should encourage motivation with the aim of creating adherence to sports practice and improving motor behaviour and its development, creating active and healthier lifestyles.

In this perspective, the Touchtennis modality has recently arrived in Spain with its first circuit organised by the Royal Tennis Federation (55). However, no evidence of its implementation in Spanish schools has been found. For this reason, it is interesting that it is integrated into new groups of practitioners, in order to get to know a new and attractive discipline. Moreover, Touchtennis has interesting aspects, as it is easy to learn and can be applied quickly and economically. Therefore, this sport can be an effective resource to introduce in the educational environment (56).

## 3 Learning environment

Touchtennis is a split-court racket sport, which maintains the essence of conventional tennis in a shortened version. This sport makes it unique, due to its striking adaptability and accessibility at an early age, making it a suitable choice for inclusion in the educational environment.

Specifically, it is a sport that is played individually or in pairs, positioned on both sides of the court and divided by a net. The main objective of the game is to win points through a rally, in which the aim is to force the opponent to make a mistake or to hit a winning shot. However, it has similarities to conventional tennis in that it focuses on a game of precision, strategy and control, rather than speed and intensity. In this sense, thanks to its particularities, it facilitates its practice being accessible to all ages regardless of the characteristics of each individual. Moreover, thanks to the modifications in the dimensions of the court, it allows the sport to be more dynamic and entertaining. In fact, a court can be installed on the vast majority of indoor and outdoor surfaces. However, one of the practical barriers that the sport can have in educational settings is the cost and accessibility of sports equipment. However, Touchtennis equipment is easily adaptable to different types of racquets and cheaper balls. As for the court, it can be played informally by adapting the court with tape or by converting it into a Pickleball court. In this way, it is a sport that can be easily adapted in schools.

For these reasons, it is characterised as an innovative and inclusive racket sport, as it encourages motivation and enjoyment towards the practice of physical activity, making it a favourable option to incorporate in education and specifically in Physical Education classes.

### 3.1 Playing field

The Touchtennis court is divided into two halves of 6 m, a reduced version of the tennis court. The official dimensions are 12 m × 5 m for singles and 12 m × 6 m for doubles, with a net height between the two halves of no less than 0.80 m. However, variations in the dimensions of the court of up to 1 m are accepted, with the aim of offering greater variety and accessibility.

### 3.2 Equipment

In terms of equipment, a ball with a uniform surface made of a foam material with a diameter of 8 cm is required, which facilitates hitting, reduces the speed and intensity on the court and minimises the impact on the player's joints.

On the other hand, the 21-inch racquet with a strung weight of 195 g and a head size of 85 inches, is characterised by its light and reduced size, favouring its handling, movement and technique of the sport.

### 3.3 Participants

Touchtennis is an inclusive discipline, so it is designed to be participatory and accessible to all those who wish to practice a new form of racket. In addition, it is an emerging discipline that seeks an initiation to the practice of a sport regardless of skill level, whether beginners or experienced players. In this way, the practice of Touchtennis facilitates the introduction of conventional tennis, but with a more motivating and attractive factor for the participant.

However, this initiative can be used in different centres, thanks to its flexibility and adaptability in any particular context or situation. It can also be adjusted to any type of age, space and material, as long as there are modifications according to the real need and context, due to the multitude of options it presents.

### 3.4 Touchtennis rules

The rules of Touchtennis are internationally regulated by Touchtennis Pro Limited (57) and integrated in the Royal Spanish Tennis Federation (56). For your enquiry, they are detailed on the official Touchtennis website (58).

### 3.5 Pedagogical format

In the following, a Touchtennis methodological sequence is proposed for the purpose of presenting and introducing a new alternative racket sport in Physical Education classes.

For this purpose, based on the methodological sequence provided by Sánchez-Alcaraz et al. (59), a game-based methodology has been applied, integrating a global strategy for teaching sport. However, another suitable methodology that could be used would be the comprehensive model or TGFU for understanding the internal logic of import.

Specifically, the proposal is designed through a structural and gradual sequence with different global practice situations. First of all, it is recommended to start with playful activities to familiarise with the sport and its materials. Subsequently, the teaching of basic strokes will be essential to learn the technique from a general approach. Next, training in basic tactics is proposed in accordance with the internal logic of the sport, followed by playing tasks that reproduce real game situations based on technical-tactical skills and, as a final product, a Touchtennis competition is proposed, in order to associate the contents learned during the methodological sequence.

**Figure 1:**
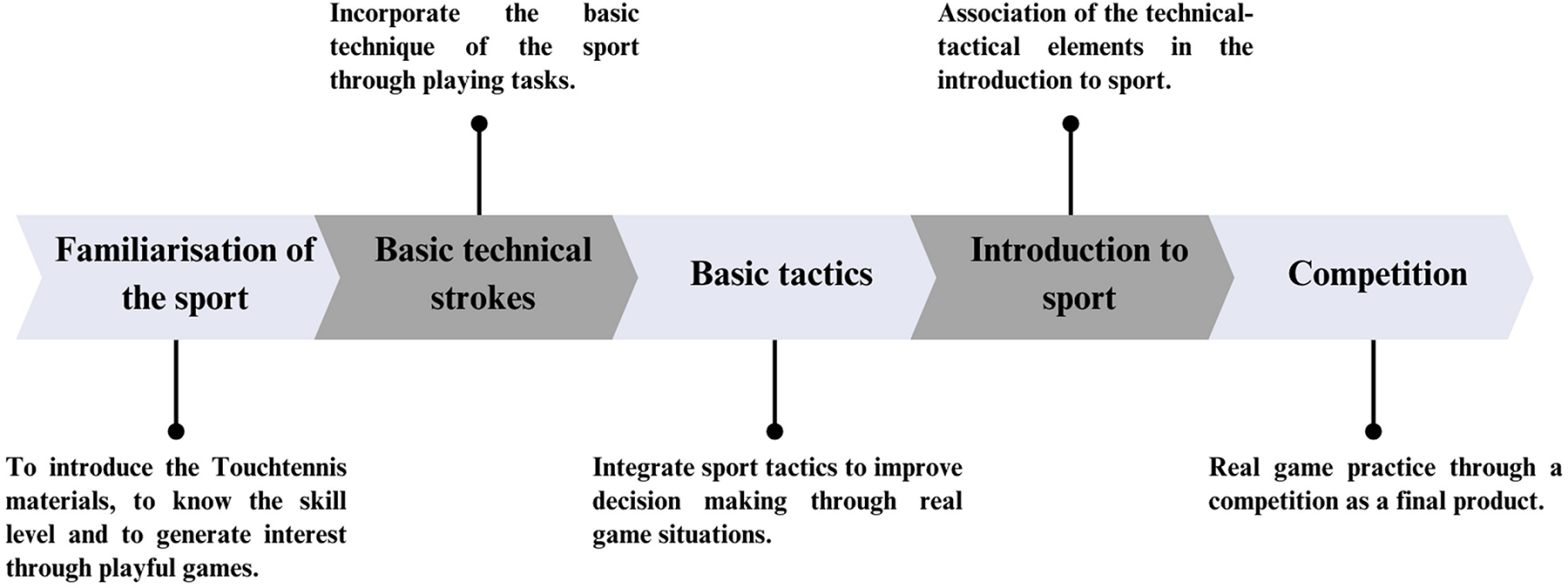
Diagram of the methodological sequence for the teaching of Touchtennis.

Consequently, it aims to increase motor engagement time and promote participation and cooperation among all students, acquiring an active role in their learning (Figure 1 and Table 1).

**Table 1:** Intervention proposal based on Touchtennis’ methodological sequence.

Methodological sequence	Didactic objectives	Task played	Graphic description
Familiarisation with the implements of the sport	To master the elements of the sport (ball, racket and racket/ball).	The introductory game consists of hitting the ball and bouncing it into one of the squares. To do this, four squares are formed and one student is placed in each of them. The objective is to cooperate among all members until a minimum number of hits is achieved. The sequence would start with hand strokes followed by racket and ball strokes.	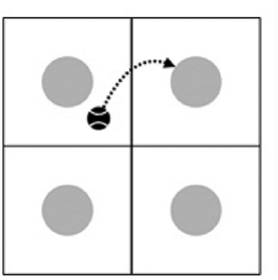
	To develop the pupils’ manipulative skills.		
	To improve the coordination aspects of the pupils.		
Learning basic strokes	To know and practice the basic technique of ground strokes (forehand and backhand), net strokes (forehand and backhand volley) and the serve.	The example of a game task based on the practice of the basic technique is based on the practice of different ground strokes, net strokes and serves to the coloured hoops distributed on the other side of the court. Each hoop will have a score and the objective will be to reach a minimum score between all members.	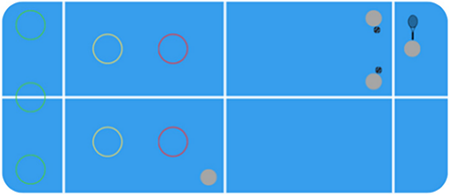
	To learn the technique from a global strategy, using the game as a methodological resource.		
Development of basic tactics	Knowing the areas of the court applying the strategy and the appropriate stroke according to the game situations (attack and defence).	In order to practise tactics, the traffic light theory is used. The game consists of a parallel rally between pairs. The aim is to prepare the point in the defence zone, force the opponent in the yellow zone and finish by hitting a winning shot in the green zone.	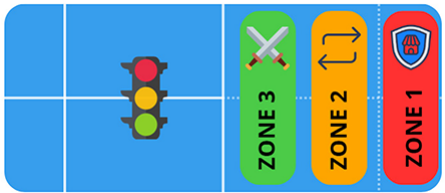
	Recognise the spatial position according to the direction of play.		
	Know the basic tactics of the sport, applying the processes of perception, decision and execution appropriate to the internal logic of the game.		
Introduction to the sport	To learn the rules in a practical way, encouraging fair play and sportsmanship among students.	The task played consists of a rally between the two pairs diagonally. Once it fails, the point will be played between the four participants. The referee will keep track of the strokes and the scoring of the game, in order to learn the Touchtennis scoring system: 15–30–40-game.	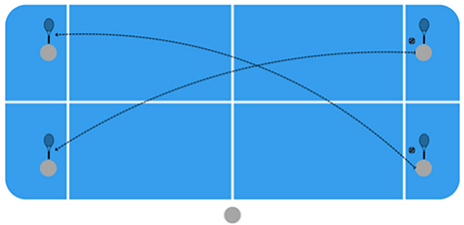
	To integrate the strokes learnt in order to internalise them, improving the precision of the strokes and the handling of the implements through a real situation.		
Final competition	Put into practice the technical-tactical aspects learnt during the methodological sequence as a final product.	In order to associate the contents, a real game situation is proposed through a Touchtennis competition. Different matches will be played on the proposed courts, the winning pair will move up a level and the losing pair will move down a level. A fair play programme will be implemented to ensure inclusion, equal opportunities and to foster a peaceful and healthy competitive spirit. To this end, the aim will be to comply with the rules, penalise unsportsmanlike conduct and harmful behaviour.	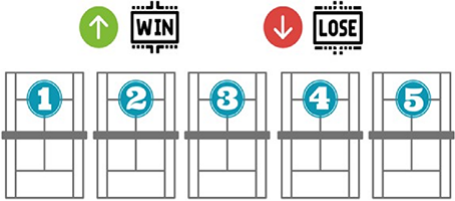

## 4 Results

Through the intervention proposal, it is intended that the schoolchildren acquire basic knowledge about Touchtennis through active, playful and meaningful learning based on the game as an educational resource. Likewise, with its dissemination, the aim is to get to know a new racket sport that encourages active participation through the application of different strategies and motor experiences during sport practice, with the aim of creating enriching experiences in the student's learning process. In addition, one of the main characteristics is that the basic technical-tactical aspects of this sport are similar to the best known racket sports, such as tennis and paddle tennis.

In a specific way, Touchtennis offers new motor proposals for sport initiation that stand out for being inclusive, providing a universal language among its practitioners. As a result, this alternative sport provides significant motor experiences, acquiring a high didactic potential and a high pedagogical quality to the educational system.

Finally, in terms of evaluation, the aim is to gather information on the student's teaching-learning process, assessing their progress and evolution in order to analyse and make a judgement on the student's performance. To this end, the procedural, conceptual and attitudinal components are evaluated, through different procedures and varied and adapted instruments, in order to know to what extent the expected results are achieved.

## 5 Discussion

The main objective of this research work was to provide the educational community with a proposal for a pedagogical intervention based on a racket sport, called Touchtennis, with the aim of inculcating these sports in educational centres in a novel and transformative way using play as an educational resource.

In relation to racket sports, Herrero et al. (23) stated that badminton and alternative sports are the most demanded by teachers in Physical Education classes. In fact, the results of Ruiz-Malagón et al. (60) showed that racket sports are appropriate for increasing motivation and decreasing anxiety in Physical Education classes. In this regard, Pradas and Castellar (61) revealed that they are considered as a key, beneficial and highly recommended content due to their multiple pedagogical facilities and possibilities.

However, Lara-Bocanegra and Galán-López (62) pointed out that it is essential that racket sports training begins in higher education, with the intention that future teachers practice and obtain extensive knowledge to implement them in their programmes.

As a result of the above, evidence supports the argument that students at any educational stage, acquire an increase in motivation and interest when including new or alternative sports, as they propose a closer and more interesting experience, away from other more recurrent proposals where the same sports disciplines are usually taught (63, 64). However, situations and strategies should be created that promote participation, increase enjoyment and interest in the subject (65) and prove to be decisive for the development of physical, cognitive, social and psychological skills (66–68).

At present, the scientific base includes a scarce amount of research related to alternative sports (69). For this reason, Pérez-Pueyo and Hortigüela (70) expressed the need to apply pedagogical interventions to discover their true contributions and move away from current trends that simply value them as different from the standard.

Moreover, these sports emerge to address the current problem of stereotypes and achieve a more egalitarian component in the subject of Physical Education (71). In fact, Hernández et al. (14) added that such sports promote integration and sportsmanship. In the same perspective, Aznar (72) underlined the importance of racket sports in terms of coeducation, equity and interculturality.

In this sense, research by Van Acker et al. (73) showed that the female gender maintained the same prominence as the male gender, and even had greater participation during practice. Therefore, implementing these sports in the educational community can have a positive impact (74) and, consequently, resolve one of the most widespread concerns in the educational context (75).

Therefore, supporting this idea, Robles and Robles (21) indicated that these sports generate greater enjoyment, unlike more traditional and habitual sports in Physical Education sessions. Likewise, the studies by Jaquete and Ramírez (76) showed that during the practice of alternative sports, more empathy and cooperation was generated among students.

In reference to the above, the findings of Menescardi and Villarrasa-Sapiña (77) showed an increase in intrinsic motivation after having practised different alternative sports in Physical Education sessions. For these reasons, its implementation is necessary as content to be taught in Physical Education classes, in addition to continuing to expand new knowledge about its benefits (19).

Specifically, the study carried out by González-Cutre and Sicilia (78) pointed out the importance of implementing novel strategies in order to obtain positive effects on adherence to physical activity. These results are in line with the research obtained by Kalajas-Tilga et al. (79), as they identified the enjoyment factor as essential and, consequently, the increase of intrinsic motivation of the schoolchildren towards the subject.

For these reasons, Touchtennis is a striking and optimal modality for incorporation into teaching programmes, which means that teachers should seek new pedagogical alternatives (17) by applying avant-garde strategies and using new experiences such as alternative sports, in order to generate improvements in motor commitment (80) and increase motivation towards the subject (20, 81).

## 6 Conclusion

Physical Education has undergone remarkable advances in its pedagogical process resulting in a high impact on education. This change has included a growing trend in the inclusion of alternative sports in its programmes, as a reflection of a need that offers new and more enriching experiences for the student. Among them, Touchtennis stands out as a sport of initiation to racket sports supported by the Royal Spanish Tennis Federation and being an unknown sport in the field of education and sport. In this sense, its impact can be attractive in the field of Physical Education, as it offers multiple advantages, including its simple learning, adaptability in the reduced and accessible material for educational centres and its enjoyment after practice as it does not require a high level of motor involvement. For these reasons, its learning introduces a new alternative sport, providing a wide variety of content in educational programmes.

The main limitation of the study is the lack of empirical data to support the pedagogical proposal. In this sense, the present study aims to create a basis for future lines of research that, through the practical application of the pedagogical proposal, will support empirical evidence and sustain a theoretical framework specific to sport. In this regard, further research is intended to analyze, firstly, the motor behavior and the impact on the physical condition of the student, through the measurement of heart rate or physical tests adapted to the sport. Secondly, the psychological and social aspects such as, for example, the level of motivation and interest caused by Touchtennis as a sport in educational programs and, finally, to analyze the didactic methodology used for an ideal development of the methodological sequence. In addition to the above, its application on a large scale at various educational levels is required, avoiding a biased sample, to verify possible differences and support the benefits of incorporating a new sporting experience in teaching programs.

For this reason, the aim is to bring an innovative practice based on a racket sport through the development of a pedagogical proposal, so that the student has the basic knowledge of an alternative sport that breaks with conventional practices and that begins to be carried out from an early age. It also promotes teamwork, mutual support, values such as fair play, sportsmanship and respect for diversity.

In short, there is a need to expand racket sports that are not very common in the educational itinerary, so that new practice options are known that enrich the sporting experience of the schoolchildren.

## Data Availability

The original contributions presented in the study are included in the article/Supplementary Material, further inquiries can be directed to the corresponding author.
